# Controlled Trial Data Casts Doubt on the Supposed Benefit of Lung Metastasectomy. Comment on Chandra et al. The Colorectal Cancer Tumor Microenvironment and Its Impact on Liver and Lung Metastasis. *Cancers* 2021, *13*, 6206

**DOI:** 10.3390/cancers14174235

**Published:** 2022-08-31

**Authors:** Fergus Macbeth, Tom Treasure

**Affiliations:** 1 Centre for Trials Research, Cardiff University, Cardiff CF14 4YS, UK; 2 Clinical Operational Research Unit, University College London, London WC1H 0BT, UK

We read with interest the comprehensive review by Chandra et al. of the biology of metastases from colorectal cancer (CRC) [1]. They describe the limitations of current approaches to the management of metastatic CRC and refer to the PulMiCC trial, the only randomised controlled trial (RCT) of surgical pulmonary metastasectomy. They say this trial ‘was stopped early due to poor recruitment’ but do not present its findings. Full results of this trial have now been published [2] and tell an important story.

The randomized trial was nested within a large careful, prospective observational study [3]. Clinical teams chose to operate on 263 patients and to not operate on 128. If teams were in equipoise about metastasectomy, and the patients consented, they were randomized into the RCT (N = 93). The overall survival (OS) of all three groups is shown in Figure 1. Those who were selected for metastasectomy had significantly longer OS than those who were not, but analysis has shown that they had a much higher rate of good prognostic factors: single metastasis, non-elevated CEA, and freedom from liver involvement. They were also younger, with better lung function and overall performance status. These differences resulting from clinical selection would account for the difference in survival. When they were balanced in the two arms of the RCT there was no difference at any time point. Although the 93 patients randomized were insufficient to prove non-inferiority, the results clearly rule out the major survival benefit that has been widely promoted. It is interesting to note that the three-year OS of the control patients was around 65% not very different from the 63.8% reported by Beppu et al [4] cited by Chandra et al. in their Table 1 [1].

These findings together with the results of the SEER database study [5] which suggests no OS benefit from pulmonary metastasectomy, need serious consideration. The NCCN guideline committee and other opinion leaders in the management of CRC should review this evidence and perhaps revise their guidance. Chandra et al. clearly indicate the opportunities for finding new targets for effective systemic treatment and this may be a better way of managing what is almost always a systemic rather than a localised clinical problem. It is perhaps now time for pulmonary metastasectomy for colorectal cancer to be consigned to history.

## Figures and Tables

**Figure 1:**
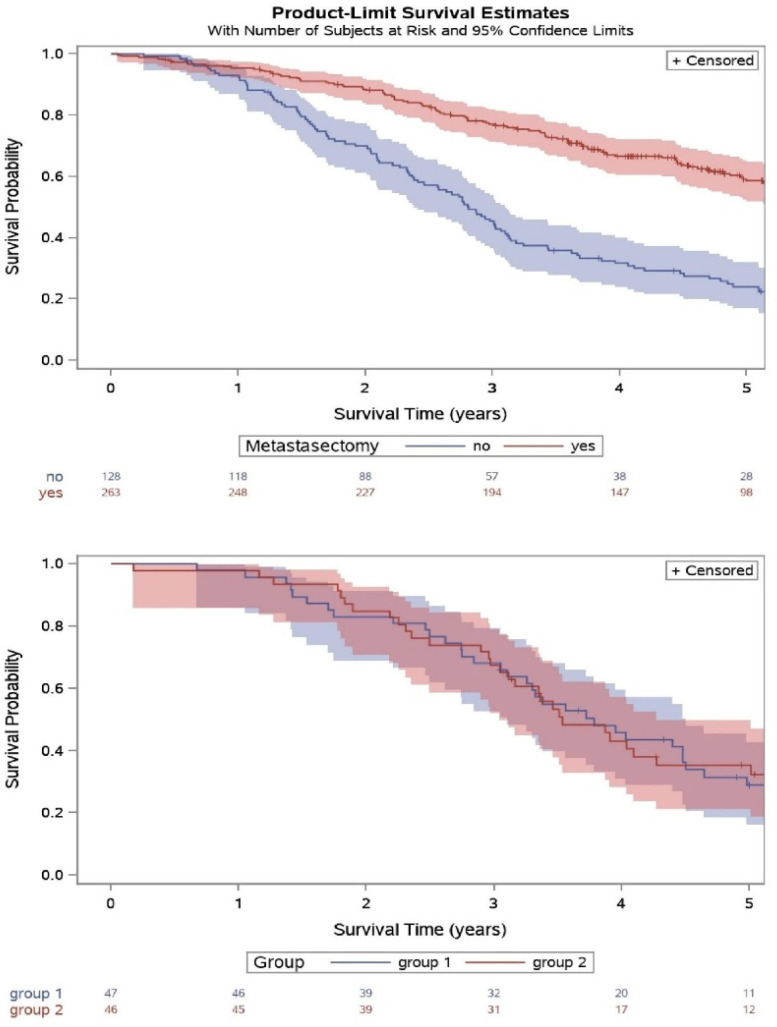
Of 484 patients with colorectal lung metastases in a prospect cohort study, with baseline and follow-up data collected to trial standards, 263 were selected for metastasectomy and 128 were not (upper panel).The survival of operated patients was comparable with the best reported “real world”ouctomes. Survival among patients not having metastasectomy was not zero or close to is as is widely assumed. In the nested controlled trial (lower panel) there was no difference between the randomly assigned arms.
